# The Effect of Curcumin-Loaded Glucan Nanoparticles on Immune Cells: Size as a Critical Quality Attribute

**DOI:** 10.3390/pharmaceutics15020623

**Published:** 2023-02-13

**Authors:** Mariana Colaço, Tiago Roquito, João Panão Costa, Maria Teresa Cruz, Olga Borges

**Affiliations:** 1 Department of Pharmaceutical Technology, Faculty of Pharmacy, University of Coimbra, 3000-548 Coimbra, Portugal; 2 Center for Neuroscience and Cell Biology, University of Coimbra, 3004-504 Coimbra, Portugal

**Keywords:** curcumin, glucan-based delivery systems, pro-oxidant properties, size-dependent cytotoxicity, hemocompatibility, immunotherapeutic agent, inflammation

## Abstract

Curcumin is known for its multiple health benefits, largely due to its antioxidant and anti-inflammatory properties. It has been extensively studied as a therapeutic agent, however, it does not have good clinical efficacy due to its poor water solubility and bioavailability. Despite accepting the encapsulation of this compound in polymeric particles as one of the most promising strategies to increase its therapeutic value, these nanoparticles have fallen short of expectations due to a lack of assessment of their possible adverse effects on the immune system. Therefore, in this work, we report on a new method to encapsulate curcumin into glucan nanoparticles and their effects on cells of the immune system were evaluated. Two different-sized curcumin-loaded glucan NPs (GluCur 100 and GluCur 380) were produced, each with an encapsulation efficiency close to 100%, and were characterized regarding their size distribution, surface properties, and morphology. The results revealed the greatest hemolytic effect and cytotoxicity for the smallest particles (100 nm) tested in human PBMCs and RAW 264.7 cells. Although GluCur 380 NPs showed a weaker ROS production, they were able to inhibit the production of NO by macrophages. Furthermore, we found that the coagulation time was not affected by both sized-particles as well as platelet function. Additionally, both nanoparticles induced lymphocyte proliferation and TNF-α secretion by Mo-DCs. In conclusion, this report emphasizes the importance of the immunotoxicity assessment and how this is dependent on the intrinsic properties of nanomaterials, hopefully contributing to increasing the safety of nanomedicines.

## 1. Introduction

The maintenance of cellular redox homeostasis is crucial for a healthy physiological condition and cell survival [1]. Unlike a normal healthy cell, cancer cells are characterized by higher levels of reactive oxygen species (ROS), which creates a state of redox imbalance. Over the last few years, many researchers have studied the potential of using this increased oxidative stress to find strategies to combat cancer. Recent studies have focused on the use of pro-oxidant compounds as polyphenols to treat several types of cancers, namely, hepatocellular carcinoma, the most common form of liver cancer [2].

Curcumin is a polyphenol obtained from a plant native to Southeast Asia, *Curcuma longa* (turmeric). In the last decade, this compound has attracted increasing attention because it is associated with a wide spectrum of biological activities such as anti-inflammatory, antioxidant and/or pro-oxidant, anti-carcinogenic, neuroprotective, and immunomodulating properties [3,4]. The anti-inflammatory properties of curcumin seem to be explained by its capability to control a variety of molecular targets related to the process of inflammation such as the enzymes lipoxygenase 5, cyclooxygenase 2, and inducible nitric oxide synthase [5], cytokines (TNF-α, IL-1β, IL-1, IL-2, IL-6, IL-8 and IL-12) [6], or transcription factors (NF-kβ) [7]. Several studies have also focused on the powerful antioxidant effect of curcumin [8,9,10]. Curcumin demonstrated the inhibition of ROS by modulating the activity of antioxidant enzymes such as catalase, superoxide dismutase, or glutathione peroxidase [11]. Nevertheless, there is evidence that curcumin also has a pro-oxidant effect [12]. It was clarified that when using lower concentrations of curcumin, this acted as an antioxidant, reducing the levels of ROS acting as a chemopreventive agent [13]. In contrast, when using higher concentrations of curcumin, a pro-oxidant effect was observed, leading to increased levels of ROS, capable of inducing cell apoptosis, which could justify the claimed chemotherapeutic effect [14]. Furthermore, curcumin can modulate cellular apoptosis or induce cell cycle arrest, which makes this compound a potent anti-carcinogenic agent. The latter effect is particularly due to the downregulation of genes involved in tumor growth and survival such as B-cell lymphoma 2 (BCL-2), BCL-xL, and the proto-oncogene c-MYC, and upregulate genes involved in apoptosis such as the bax protein, BCL-xs, and p53 protein [15]. In terms of immunomodulating effects, curcumin can activate cells as macrophages, natural killer cells, and T and B cells, and it was even reported to minimize T-cell apoptosis induced by tumors [16].

Curcumin presents a good toxicity profile, being considered quite tolerated by humans. This has been shown in clinical studies, where daily doses of 8 g were administered, and no significant side effects were detected. Furthermore, this compound has already been approved by the Food and Drug Administration (FDA) as a safe curcuminoid [17]. Despite the safety profile and applications of curcumin, the clinical application is quite limited. Several reports have attributed this fact to curcumin reduced bioavailability. In a clinical trial with 15 patients, the participants received daily oral curcumin doses between 450 mg and 3600 mg. The authors of the study concluded that with the highest dose, it only detected low levels of the drug, in the order of 0.63 ng/mL in plasma [18]. The reasons for this low bioavailability are curcumin poor water solubility, high degradability at physiological pH, poor absorption, and the fact that curcumin is quickly metabolized [19]. In terms of stability, curcumin has been shown to decompose very rapidly in basic conditions, changing its yellow color to a dark red. Nonetheless, in acidic conditions, the stability of this polyphenol is improved [20]. 

To overcome these difficulties, several solutions have been tried and described. For instance, the association with other compounds decreases the rapid excretion of curcumin, improving its metabolic pathway efficiency. One of those examples is piperine, a constituent of pepper, that, when administered orally in humans and combined with curcumin, it increased the bioavailability of this last compound by 2000% [21]. The use of nanocarriers such as polymeric nanoparticles [22], micelles [23], liposomes [24], nanosponges [25], nanosuspensions [26], or even biodegradable microspheres [27] to encapsulate and protect curcumin from being rapidly metabolized have also been implemented with the aim to increase the bioavailability of curcumin.

Natural polymers are materials commonly used to produce nanoparticles. Depending on the polymer chosen, these nanoparticulate systems offer low toxicity, high loading capacity, and improved stability of the drug by protecting it from degradation and by modifying its clearance [28]. Particularly, glucan has been explored in the last few years due to its immunological effect. There are several glucans, with the β-glucans one of the most immunologically relevant type, whose natural source is the cell walls of bacteria, fungi, and cereals [29]. These polysaccharides exist in distinct forms due to the different possibilities of chemical bonding between glucose molecules [30]. Curdlan, a type of 1,3-β-glucan, has been associated with immunostimulant and pro-inflammatory properties as well as an anticarcinogenic effect [31]. This is in part due to curdlan’s ability to be recognized by some pattern recognition receptors such as dectin-1, complement receptor 3, and CD5, which are present on the surface of various immune cells [32]. The binding to these receptors then triggers several effects that lead to the activation of the cells of the immune system, namely, cytokine secretion and cell maturation/differentiation/activation. The β-glucans pro-oxidant activity contributes to the claimed anticancer effects. Curdlan enhances the phagocytic ability of macrophages, that then became activated and consequently, a respiratory burst leading to the formation of reactive nitrogen and oxygen species occurs [33].

Hence, the choice of polymer is a determinant factor since it is important to have a clear understanding of the effect of the polymer-based nanocarrier on the cells of the immune system to implement the safe-by-design concept [34]. The nanocarrier can stimulate the cells of the immune system and this effect is desirable, which can be the case of nanocarriers in chemotherapy or vaccines, or the effect is not desirable and may contribute to adverse effects of the medicine. For example, it does not seem desirable to stimulate the cells of the immune system when nanoparticles carry therapeutic proteins (like insulin), because we would not want to produce antibodies against that therapeutic protein. 

The nanoparticle (NP) size determines the kinetics and the efficiency of cellular uptake as well as its toxic potential for humans [35]. Furthermore, the mechanism of entrance in the cell may also differ. It is known that small nanoparticles, with sizes up to 150 nm, enter the cells via clathrin- or caveolin-mediated endocytosis and have significant biological activity. Conversely, particles with a size range of 250 nm to micrometers have been shown to enter cells via a phagocytosis process, and have associated longer-lasting effects [36]. For this reason, it is crucial to consider the size of the particle when developing a drug nanocarrier. This characteristic represents a critical quality attribute (CQA) of manufactured nanomaterials [37].

This study had two objectives: (1) To develop a method to encapsulate curcumin into two sized-glucan particles, and (2) study the influence of the characteristics of the particles such as size and composition on the activity of the immune cells. 

The immunotoxicity studies were completed using diverse cell models: human whole blood, peripheral blood mononuclear cells (PBMCs), monocyte-derived dendritic cells (Mo-DCs), and a murine macrophage cell line (RAW 264.7 cells). Hemocompatibility (hemolysis, platelet aggregation, and coagulation time assays) was performed considering the appropriate controls.

## 2. Materials and Methods

### 2.1. Materials

Curdlan (P-Curdl lot 60201) was purchased from Megazyme (Bray, Ireland). Curcumin (C1386-5G), thiazolyl blue tetrazolium bromide (MTT), lipopolysaccharide from Salmonella enterica Serovar Minnesota (LPS), Dulbecco’s modified Eagle medium (DMEM), Roswell Park Memorial Institute (RPMI 1640) medium, HEPES buffer, heat-inactivated fetal bovine serum (FBS), and the murine cell line RAW 264.7 were obtained from Merck Life Sciences (Algés, Portugal). Sartorius™ Vivaspin™ 20 centrifugal concentrator (MWCO 300 kDa) and H_2_DCFDA (2′,7′-dichlorodihydrofluorescein diacetate) were purchased from Thermo Fisher Scientific Inc. (Waltham, MA, USA). Apyrogenic water was kindly provided by Fresenius Kabi (Tondela, Portugal). Human IL-6, IL-10, IL-12, TNF-α, and murine IL-1β ELISA development kits were purchased from PeproTech (London, UK). The other chemicals and reagents used were from normal suppliers of analytical grade.

### 2.2. Particle Production

Particle production was based on a nanoprecipitation method in which curcumin was encapsulated in two different-sized glucan particles (GluCur 100 and GluCur 380). GluCur 100 NPs were produced by adding 6.5 mL of a solution of 8% acetic acid to a 0.025% glucan solution in 2% sodium hydroxide and 1% Tween 80^®^ under magnetic stirring until reaching a pH of 11. After that, it was introduced dropwise 2.5 mL of the same solution of acetic acid but containing 0.1 mg/mL of curcumin. This addition was interrupted at different pH, namely 7, 6, and 5 for half an hour and kept under magnetic stirring. GluCur 380 NPs were obtained by the dropwise addition of a solution of 8% acetic acid with curcumin (0.1 mg/mL) to 5 mL of 0.1% glucan solution in 2% sodium hydroxide and 0.2% Tween 80^®^ under magnetic stirring. Before introducing curcumin to prepare the GluCur 380 NPs, a solution of 4% acetic acid was added until pH 11 to avoid curcumin degradation. The final suspension of nanoparticles (pH 5) was kept for 1 h under magnetic stirring to achieve NP maturation. To produce NP controls (curcumin-unloaded glucan particles called Glu 130 and Glu 355), a similar procedure was implemented but without adding the drug to the solution of acetic acid, as already described by our group [38]. The two different systems of nanoparticle suspensions were concentrated and washed with pyrogen-free water to remove the original solvents using Vivaspin 20 centrifugal concentrator (MWCO 300 kDa).

### 2.3. Particle Characterization

#### 2.3.1. Size and Zeta Potential Measurements

The mean hydrodynamic diameter of the curcumin-loaded glucan nanoparticles was measured by dynamic light scattering (DLS) in the original solvent and in water using a Delsa^TM^ Nano C particle analyzer (Beckman Coulter, Brea, CA, USA), and the zeta potential (ZP) was measured in water using a Zetasizer Nano ZS (Malvern Instruments, Ltd., Worcestershire, UK) through electrophoretic light scattering (ELS).

#### 2.3.2. Estimation of (1-3)-β-Glucan Content in the NPs

To quantify the incorporation of glucan into curcumin-loaded glucan NPs, the polymer was measured in the supernatants of the centrifuged NPs and measured using the fluorescence dye-binding microassay, a method already described elsewhere [39]. Briefly, 300 µL of the supernatants of the NPs were added to 30 µL of 6 N sodium hydroxide (NaOH). The samples were left incubating for 30 min at 80 °C and were then immediately transferred to an ice bath before the addition of 630 µL of a dye solution (40 mL of 0.1% aniline blue in water, 21 mL of 1 N HCl, and 59 mL of 1 M glycine/NaOH buffer, pH 9.5). After that, the samples were incubated at 50 °C for 30 min and subsequently incubated for another 30 min at room temperature to decolorize the unbound fluorescent dye. The fluorescence was measured at an emission wavelength of 502/20 nm and an excitation wavelength of 398/20 nm. A calibration curve ranging from 0 to 24 µg/mL was conducted using a series of diluted glucan concentrations in 1 N NaOH to later quantify the free glucan concentration by interpolation of the values.

#### 2.3.3. Quantification of Curcumin Loading Efficacy in Curcumin-Loaded Nanoparticles

After both types of curcumin-loaded glucan NPs (GluCur 100 and GluCur 380) were concentrated, the filtrate was used to quantify the free curcumin not encapsulated into the nanoparticles. For that, a calibration curve was prepared using known quantities of curcumin dissolved in the solvent of NPs (acetic acid + NaOH) and the samples were read at a maximum wavelength of 469 nm. After drawing the calibration curve, the quantity of free curcumin was determined.

#### 2.3.4. Assessment of Physicochemical Stability of Curcumin-Loaded Glucan NPs at Two Different Temperatures

The GluCur 100 and GluCur 380 NPs were concentrated and washed with pyrogen-free water and individual batches of each NP were stored at 4 °C and 20 °C for 6 weeks to assess the nanoparticle stability. The resulting size and polydispersity index (PDI) of the particles were measured using a Delsa^TM^ Nano C particle analyzer (Beckman Coulter, Brea, CA, USA) and zeta potential through Zetasizer Nano ZS (Malvern Instruments, Ltd., Worcestershire, UK) at different time points. 

### 2.4. Hemocompatibility Studies

#### 2.4.1. Hemolysis Assay

Human whole blood samples were collected from volunteer healthy donors in heparinized tubes after all subjects gave their written informed consent in accordance with the Declaration of Helsinki. The blood was diluted in PBS to adjust the total hemoglobin concentration to 10 mg/mL ± 2 mg/mL (dTBH). A sample of 100 µL of curcumin-loaded glucan NPs, free curcumin, Triton-X-100 (positive control), and PBS (negative control) were added to 700 µL of PBS and 100 µL of the diluted blood (dTBH) in different microtubes. Blank NPs were also tested in a dilution equivalent to curcumin-loaded glucan NPs as the vehicle controls. To determine the possible NP interference with the assay, GluCur 100 and GluCur 380 NPs were incubated with PBS, without blood. The samples were incubated at 37 °C for 3 h and then centrifuged at 800× *g* for 15 min. In a 96-well plate, 100 µL of the supernatants of each sample was added, as was 100 µL of cyanmethemoglobin (CMH) reagent. The absorbance (OD) was measured at 540 nm using a microplate reader and the percentage of hemolysis was calculated by the following equation:

(1) $$\mathrm{Hemolysis}\;\left(\%\right)=\frac{\left(\mathrm{OD}\;\mathrm{sample}\;-\;\mathrm{OD}\;\mathrm{negative}\;\mathrm{control}\right)}{\left(\mathrm{OD}\;\mathrm{positive}\;\mathrm{control}\;-\;\mathrm{OD}\;\mathrm{negative}\;\mathrm{control}\right)}\times 100$$

#### 2.4.2. Coagulation Time Assay

The extrinsic and intrinsic pathways of blood coagulation were separately tested by assessing the prothrombin time (PT) and activated partial thromboplastin time (APTT), respectively. Blood was collected in tubes with sodium citrate as an anticoagulant and later centrifuged at 2500× *g* for 10 min to obtain the human plasma. A sample of 450 μL of plasma was incubated with 50 μL of curcumin-loaded nanoparticles or free curcumin (final concentration 3.4 µg/mL) for 30 min at 37 °C. Then, the samples were assessed using a Bio-TP LI Kit that evaluates PT and a Bio-CK Kit that evaluates APTT, according to the manufacturer’s instructions, in an Option 4 plus coagulation analyzer (BioMérieux, Marcy-l’Étoile, France).

#### 2.4.3. Platelet Aggregation Assay

Whole blood collected in sodium citrate tubes was centrifuged at 200× *g* for 16 min to obtain the platelet-rich plasma (PRP). The platelet-free plasma (PFP) was obtained after first blood centrifugation at 2500× *g* for 10 min, succeeded by a second platelet-poor plasma (PPP) centrifugation at 18,000× *g* for 5 min. In a 96-well plate, 100 µL of PRP or 100 µL of PFP was added and incubated at 37 °C for 5 min. Thereafter, 25 µL of curcumin-loaded glucan NPs (3.4 µg/mL), free curcumin (3.4 µg/mL), collagen (positive control), and saline solution (negative control) were added to the wells with PRP and left to incubate at 37 °C for 30 min. Curcumin-loaded glucan NPs were also incubated with PFP to evaluate the NP interference in human plasma. Then, 4 µL of Giemsa dye was added to each well, and incubated for 5 min at 37 °C. The samples were visualized in the microscope and a 1:200 dilution was performed for platelet counting (PC) using a Neubauer chamber. The percentage of platelet aggregation was calculated using Equation (2):

(2) $$\mathrm{Platelet}\;\mathrm{aggregation}\;\left(\%\right)=\frac{\left(\mathrm{PC}\;\mathrm{negative}\;\mathrm{control}\;-\;\mathrm{PC}\;\mathrm{sample}\right)}{\mathrm{PC}\;\mathrm{negative}\;\mathrm{control}\;}\times 100$$

### 2.5. In Vitro Studies with Human Peripheral Blood Mononuclear Cells

#### 2.5.1. PBMCs Isolation by Density Gradient

Buffy coats obtained from healthy donors were kindly given by IPST, IP (Coimbra, PT). PBMCs were isolated through a density gradient centrifugation using Lymphoprep (Axis-Shield, Dundee, Scotland), according to the manufacturer’s guidance protocol, with minor modifications. In brief, blood was diluted in 0.9% sodium chloride (1:1.5 dilution), and the first centrifugation was performed at 1190× *g* for 20 min (20 °C). After collecting the mononuclear cell ring, a series of consecutive centrifugations were conducted at 350× *g* for 10 min (20 °C) using sterile PBS (pH = 7.4 at 37 °C) until the supernatant was clear. To remove the red blood cells, the pellet was resuspended in 5 mL ammonium-chloride-potassium lysing (ACK) buffer (150 mM NH_4_Cl, 10 mM KHCO_3_, and 0.1 mM Na_2_EDTA, adjusting the pH to 7.2–7.4) and incubated for 10 min at 4 °C. Then, 5 mL of PBS was added, and the cells were centrifuged at 300× *g* for 10 min at 20 °C. Cells were washed twice with PBS to remove the ACK buffer (10 min, at 300× *g*, 20 °C). Finally, isolated human PBMCs were cultured in RPMI 1640 with 10% heat-inactivated FBS, supplemented with 1% penicillin/streptomycin and 2 mM L-glutamine.

#### 2.5.2. Nanoparticle Cytotoxicity 

Curcumin-loaded glucan NPs and free curcumin cytotoxicity were evaluated on human PBMCs using the MTT tetrazolium reduction assay. Cells were seeded in 96-well plates at a density of 7.5 × 10^6^ cells/well. Serial dilutions of curcumin associated with GluCur 100, curcumin associated with GluCur 380 NPs, and free curcumin were prepared at a final concentration range between 0.27 µg/mL and 25 µg/mL in the well and were incubated with the cells for 24 h at 37 °C. Blank NPs were also tested in a dilution equivalent to curcumin-loaded glucan NPs as the vehicle controls. Then, 20 µL of MTT solution (5 mg/mL) was added to each well and incubated for an additional 4 h. The 96-well plates were then centrifuged at 800× *g* for 25 min and the cell culture medium was replaced with dimethyl sulfoxide (DMSO) to ensure the dissolution of the formazan crystals. The absorbance values (OD) were measured at 540 nm and 630 nm using a microplate reader. Cell viability (%) was calculated using Equation (3):

(3) $$\mathrm{Cell}\;\mathrm{viability}\;\left(\%\right)=\frac{\left(\mathrm{OD}\;\mathrm{sample}\left(540\mathrm{nm}\right)-\mathrm{OD}\;\mathrm{sample}\left(630\mathrm{nm}\right)\right)}{\left(\mathrm{OD}\;\mathrm{control}\left(540\mathrm{nm}\right)-\mathrm{OD}\;\mathrm{control}\left(630\mathrm{nm}\right)\right)}\times 100$$

#### 2.5.3. Cell Proliferation Assay

Cell proliferation was assessed using the MTT tetrazolium reduction assay. PBMCs were incubated in 96-well plates for 2 h at 37 °C and 5% CO_2_, at a cell density of 7.5 × 10^6^ cells/well. After that, serial dilutions of curcumin associated with GluCur 100 NPs (0.02 µg/mL–1.1 µg/mL), curcumin associated with GluCur 380 NPs, and free curcumin (0.07 µg/mL–4.3 µg/mL) were incubated with the cells in RPMI for 72 h at 37 °C and 5% CO_2_. Blank NPs were also tested in a dilution equivalent to curcumin-loaded glucan NPs as the vehicle controls. The proliferation was then measured using the MTT assay, in which 20 µL of this reagent (5 mg/mL in PBS pH = 7.4) was added to each well and incubated for 4 h at 37 °C. Cell culture plates were centrifuged at 800× *g* for 25 min and the cell culture medium was replaced by DMSO. The absorbance values (OD) of the resultant colored solution were measured at 540 nm and 630 nm using a microplate reader. Cell viability (%) was calculated using Equation (3).

### 2.6. Generation of Mo-DCs and Cytokines’ Assessment

Human blood-derived monocytes were isolated using the MiniMACS^TM^ Separator and Starting Kit with magnetic human CD14 MicroBeads (Miltenyi Biotec, Bergisch Gladbach, Germany), following the manufacturer’s protocol. In 6-well plates, 1.3 × 10^6^ monocytes were incubated in 3 mL of supplemented RPMI for DCs (RPMI 1640, 10% FBS, 2 mM L-glutamine, 25 mM HEPES, 100 U/mL penicillin, 100 μg/mL streptomycin, 0.1 mM MEM NEAA, and 1 mM sodium pyruvate) with 50 ng/mL IL-4 and 40 ng/mL GM-CSF. On day 3, the medium on each well was gently mixed, and 1.5 mL of each well was removed to a 15 mL sterile tube. The cells were centrifuged at 400× *g* for 5 min at 20 °C, and the pellet was resuspended in 1.5 mL of supplemented RPMI for DCs with two times the concentration of IL-4 and GM-CSF. The cells were then added again to the original culture. On day 6, the cells were collected in a sterile 15 mL tube and each well was washed twice with PBS. The washes were added to the same tube and centrifuged at 400× *g* for 5 min at 20 °C. Immature dendritic cells were seeded in 24-well plates at a density of 1.5 × 10^5^ cells/well and 500 µL of the stimulus was added and incubated for 24 h. Moreover, the immature dendritic cells were phenotypically evaluated by labeling with the surface markers CD14 and CD11c using a BD Accuri^TM^ C6 Plus cytometer. The supernatants were collected into plates and kept at −80 °C for the assessment of the presence of pro- and anti-inflammatory cytokine secretion such as TNF-α, IL-6, IL-12, and IL-10. To measure these cytokines, we used the enzyme-linked immunosorbent assay (ELISA), according to the manufacturer’s instructions (Peprotech, NJ, USA). Furthermore, the Alamar Blue assay was conducted to evaluate the cytotoxicity of the used concentrations of the different formulations.

### 2.7. In Vitro Studies with a Murine Macrophage Cell Line (RAW 264.7)

The RAW 264.7 cell line was cultured in DMEM supplemented with 10% heat-inactivated FBS, 3.7 g/L sodium bicarbonate, 10 mM HEPES, and 1% penicillin/streptomycin and used until passage 18.

#### 2.7.1. Nanoparticle Cytotoxicity

The cytotoxicity of both the NPs, curcumin-loaded glucan NPs, and free curcumin was assessed by a similar protocol of the MTT assay, as described previously for PBMCs (see Section 2.5.2) with some modifications. In brief, RAW 264.7 cells were seeded in 96-well plates at a density of 2 × 10^4^ cells per well. The NP suspensions and free curcumin were tested in the same concentration ranges as in the cytotoxicity assay performed with human PBMCs and the incubation time with MTT was 90 min.

#### 2.7.2. Nitric Oxide Production Assay

Nitric oxide (NO) is extremely unstable and degrades easily to nitrites, which are products that can be spectrophotometrically determined. Thus, the NO production by RAW 264.7 cells was evaluated by the quantification of nitrites using Griess reagent (mixed 1% (*w*/*v*) sulfanilamide in 2.5% (*v*/*v*) phosphoric acid and 0.1% (*w*/*v*) naphthylethylenediamine dihydrochloride in 2.5% (*v*/*v*) phosphoric acid in a ratio of 1:1). In brief, the RAW 264.7 cells were seeded in 48-well plates at a density of 2.25 × 10^5^ cells per well for 24 h at 37 °C and 5% CO_2_. Thereafter, the cell culture medium was replaced by serial dilutions of free curcumin (0.19 µg/mL–13 µg/mL), curcumin associated with GluCur 100 NPs (0.19 µg/mL–0.75 µg/mL), and curcumin associated with GluCur 380 NPs (2.8 µg/mL–11 µg/mL) diluted in DMEM without phenol red. Blank NPs were also tested in a dilution equivalent to curcumin-loaded glucan NPs as the vehicle controls. LPS solution (1 μg/mL) was used as the positive control. To assess whether the NPs were able to inhibit the production of NO, the same free curcumin and curcumin-loaded and -unloaded glucan NP concentrations were incubated with cells in the presence of LPS (1 μg/mL). After 24 h of incubation, 100 µL of each cell supernatant was harvested and combined with an equal volume of Griess reagent in 96-well plates. In the same plate, a calibration curve of serial dilutions of sodium nitrite (0 μM to 80 μM) was also performed in duplicate. The absorbance of the samples was then measured at 550 nm using a microplate reader, and nitrite quantification was extrapolated from the calibration curve. The percentage of stimulation or inhibition of NO production was calculated by the following equation:

(4) $$\mathrm{NO}\;\mathrm{production}\;\left(\%\right)=\frac{\mathrm{NO}\;\left(µg/\mathrm{mL}\right)\;\mathrm{sample}}{\mathrm{NO}\;\left(µg/\mathrm{mL}\right)\;\mathrm{positive}\;\mathrm{control}}\times 100$$

The assay was carried out under conditions of cell viability greater than 70%.

#### 2.7.3. Reactive Oxygen Species Production Assay

ROS production was evaluated using the dichlorofluorescein diacetate probe (DCFH-DA). Briefly, RAW 264.7 cells were seeded in black 96-well plates for 24 h at 37 °C and 5% CO_2_, at a cell density of 5 × 10^4^ cells per well. Then, serial dilutions of free curcumin (0.19 µg/mL–11 µg/mL), curcumin associated with GluCur 100 NPs (0.19 µg/mL–0.75 µg/mL), and curcumin associated with GluCur 380 NPs (2.8 µg/mL–11 µg/mL) diluted in DMEM were incubated with the cells. Blank NPs were also tested in a dilution equivalent to curcumin-loaded glucan NPs as the vehicle controls. The LPS solution (1 μg/mL) was used as the positive control. To assess whether the NPs were able to inhibit the production of ROS, the same free curcumin and curcumin-loaded and -unloaded glucan NP concentrations were incubated with cells in the presence of LPS (1 μg/mL). After 24 h of incubation, the cell culture medium was removed and replaced by a DCFH-DA probe (50 μM) previously dissolved in serum-free DMEM. The cells were left incubating for an additional 2 h at 37 °C, 5% CO_2_, and the resulting fluorescence was measured at 485/20 (excitation wavelength) and 528/20 (emission wavelength). The percentage of the stimulation or inhibition of ROS production was calculated by applying the following equations:

(5) $$\mathrm{ROS}\;\mathrm{production}\;\left(\%\right)=\frac{\mathrm{Fluorescence}\;\mathrm{sample}}{\mathrm{Fluorescence}\;\mathrm{negative}\;\mathrm{control}}\times 100$$

(6) $$\mathrm{ROS}\;\mathrm{inhibition}\;\left(\%\right)=\frac{\mathrm{Fluorescence}\;\mathrm{sample}}{\mathrm{Fluorescence}\;\mathrm{positive}\;\mathrm{control}}\times 100$$

The assay was carried out under conditions of cell viability greater than 70%.

#### 2.7.4. Cytokine Secretion

RAW 264.7 cells were plated in 48-well plates at a density of 2.25 × 10^5^ cells/well and incubated with free curcumin (0.19 µg/mL–11 µg/mL), curcumin associated with GluCur 100 (0.19 µg/mL–0.75 µg/mL), and curcumin associated with GluCur 380 (2.8 µg/mL–11 µg/mL) to determine the effect of the formulations on cell cytokine secretion. A positive control, LPS (1 μg/mL), and blank glucan NPs were also incubated with the cells for 24 h at 37 °C and 5% CO_2_. After that period, cell culture plates were centrifuged at 800× *g* for 25 min (20 °C) and the supernatants were removed and stored at −80 °C until the analysis of IL-1β secretion. To measure this cytokine, we used an enzyme-linked immunosorbent assay (ELISA) according to the manufacturer’s instructions (Peprotech, NJ, USA). The assay was carried out under conditions of cell viability greater than 70%.

### 2.8. Statistical Analysis

Data were analyzed using GraphPad Prism 5 (GraphPad Software, Inc., La Jolla, CA, USA), in which significant differences were obtained from one-way ANOVA Dunnett (comparison of all columns vs. control column) and the values were considered statistically different when *p* < 0.05. Results were expressed as the means ± standard error of the mean (SEM).

## 3. Results

### 3.1. Lower Concentration of Curdlan during NP Preparation Leads to Smaller-Sized and Highly Stable Curcumin-Loaded NPs

**Figure 1:**
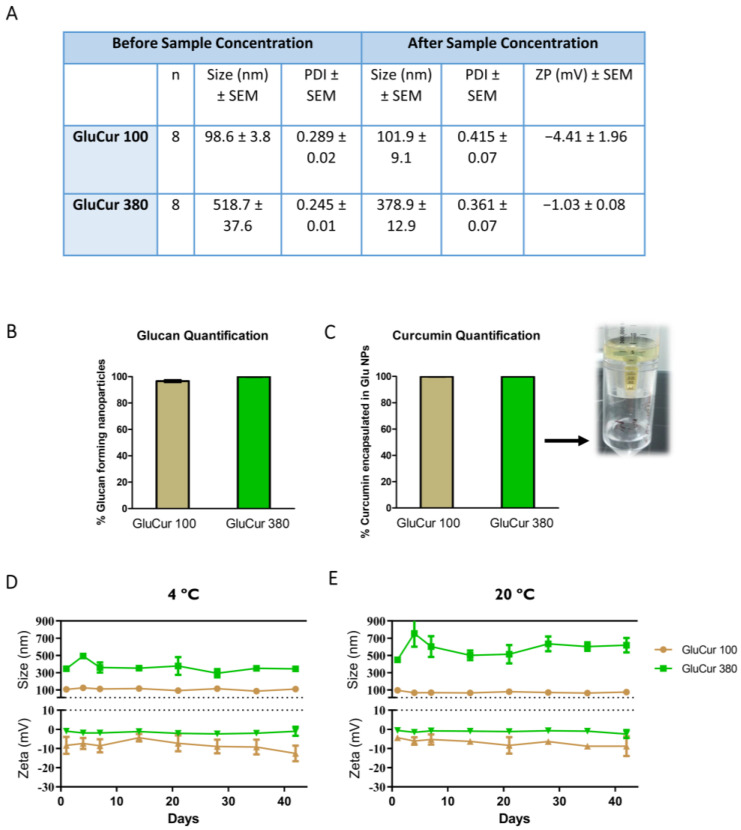
Physicochemical characterization of curcumin-loaded glucan NPs. (**A**) the mean size distribution, polydispersity index, and zeta potential were measured before (in the initial medium) and after concentration (in pyrogen-free water) of the particles. Data are presented as the mean ± SEM, n = 8 (eight independent experiments, each in triplicate). (**B**) Estimation of 1,3-β-glucan content that was used to produce curcumin nanocarriers through the aniline blue fluorescence microassay. (**C**) Quantification of curcumin using a calibration curve of known concentrations of the drug in the solvent of NPs via a UV–Visible spectrophotometer. Illustrative image of the filtrate obtained from the centrifugation of NPs with curcumin encapsulated using Vivaspin (lower part of the centrifugal tube). Assessment of the temperature effect at 4 °C (**D**) and 20 °C (**E**) on the stability of GluCur 100 and GluCur 380 NPs by measuring the size and zeta potential over 42 days. Data are presented as the mean ± SEM, n = 3 (three independent experiments, each in triplicate).

Knowing that different NP properties such as size may lead to entirely different immunotoxicity profiles [40], a complete characterization of the nanomedicines is crucial before starting the immunotoxicity studies. The nanoparticles were prepared with curdlan (1,3-β-d-glucan isolated from Alcaligenes faecalis) through a nanoprecipitation method. The preparation method was designed knowing that the polymer was soluble in alkaline solutions and insoluble in acidic environments. Thus, curdlan was first dissolved in NaOH and then precipitated with acetic acid until reaching pH 5. To obtain the two different-sized NPs, curdlan concentration was varied: 0.025% for GluCur 100 NPs and 0.1% for GluCur 380 NPs. For the encapsulation of curcumin into the NPs, a small variation in the blank NP method was introduced. First, the basic glucan solution (around pH 13) was partially neutralized with the 8% acetic acid solution and after reaching pH 11, the acetic acid solution containing curcumin was added. The pH 11 was chosen because it is close to the turning point (in only one or two drops the solution passed from pH 11 to 7), and consequently, we could be sure that the stability of curcumin was not affected. This is because curcumin is a compound that is easily degraded in extreme basic environments [41]. This procedure was applied for both sized curcumin-loaded glucan NPs. After the GluCur 100 and GluCur 380 NPs were produced, they were characterized. The mean diameter, PDI, ZP, and morphology are illustrated in Figure 1. The mean diameter of GluCur 100 NPs obtained in the original solvent was 98.6 ± 3.8 nm while for the GluCur 380 NPs, it was 518.7 ± 37.6 nm. After the washing process with apyrogenic water and the concentration step, the size of the GluCur 380 NPs decreased to a mean diameter of 378.9 ± 12.9 nm, possibly due to the disappearance of some aggregates. Likewise, the zeta potential of both NPs when dispersed in pyrogen-free water was quite similar and close to neutrality (−4.41 mV for GluCur 100 and −1.03 mV for GluCur 380) (Figure 1A).

By analyzing the supernatants of the GluCur 100 and GluCur 380 NPs after being concentrated with Vivaspin, it was possible to estimate that almost all curdlan (approximately 99% for GluCur 100 and 100% for GluCur 380 NPs) was used during the production of the NPs (Figure 1B). Additionally, the supernatant analysis also indirectly revealed that all curcumin was encapsulated in the NPs (Figure 1C).

To assess the physical stability of the NPs, stability studies were performed at two different temperatures (4 °C and 20 °C) for 42 days. The GluCur 100 NPs were demonstrated to be quite stable, regardless of the storage temperature, by maintaining their size and ZP during the experimental assay (Figure 1D,E). In contrast, the GluCur 380 NPs exhibited a greater tendency to form aggregates, predominantly at 20 °C, after only one week, revealed by a 200 nm increase in the NP size (Figure 1E). The ZP remained the same.

### 3.2. GluCur 380 NPs Present a Better Hemocompatibility Profile Than the Smaller-Sized GluCur 100 NPs

**Figure 2:**
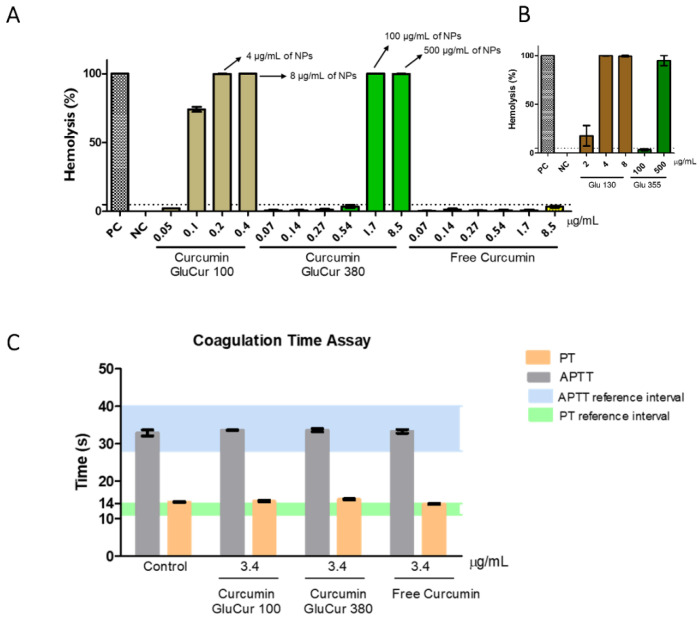
Interaction of curcumin nanocarriers with blood components. (**A**) Human red blood cell lysis (%) induced by several concentrations of curcumin associated with glucan NPs and free curcumin ranging from 0.05 to 8.5 µg/mL in human whole blood after 3 h of incubation at 37 °C. Triton-X-100 and PBS were used as the positive (PC) and negative control (NC), respectively (mean ± SEM, n = 3, three independent experiments, each in duplicate). (**B**) Hemolytic effect of blank glucan NPs in the same particle concentrations as curcumin-loaded NPs (for Glu 130—2, 4, 8 µg/mL and for Glu 355—100, 500 µg/mL) in human whole blood. (**C**) Effect of curcumin and curcumin associated with NPs at 3.4 µg/mL on blood coagulation time after 30 min incubation with platelet-poor plasma. PBS was used as a negative control. The intrinsic and extrinsic coagulation pathways were independently tested by measuring PT (reference range 11–14 s) and APTT (reference range 28–40 s). Results are expressed as the mean ± SEM from at least three independent experiments.

Hemolysis is the term used when the damage of red blood cells occurs with the consequent release of intracellular proteins, which can lead to anemia and renal failure [42]. To determine the hemocompatibility of a nanomaterial, it is crucial to assess the hemolytic ability of the NPs, particularly if it is being considered the intravenous route of administration [43]. Therefore, curcumin-loaded glucan NPs were incubated with human whole blood for 3 h at 37 °C. According to the ASTM E2524-08 standard, only values above 5% are considered hemolytic [44]. The results showed that GluCur 380 NPs were more biocompatible than GluCur 100 NPs. These smaller particles, for concentrations above 0.1 µg/mL of encapsulated curcumin, which corresponds to 2 µg/mL of NPs, showed a hemolytic percentage of almost 100% (Figure 2A). For the GluCur 380 NPs, high hemolytic activity was only observed for concentrations equal or greater to 1.7 µg/mL of encapsulated curcumin, which corresponded to 100 µg/mL of NPs. Free curcumin was also tested in the same concentrations of the encapsulated curcumin and it did not cause hemolysis (values inferior to 5%) by itself, which indicate that the toxicity came from the nanoparticulate delivery system. To validate this statement, we also analyzed the results obtained for blank NPs (Glu 130 and Glu 355) and it was indeed confirmed that the observed hemolytic activity for the curcumin-loaded glucan NPs was associated with the vehicle, as previously reported by our group [38], and not with the drug (Figure 2B). Although a synergism between the curdlan NP and the loaded-drug seemed to occur in both the GluCur 100 and GluCur 380 NPs, this is because the system became more toxic than just the NP itself.

The hemostatic balance is fundamental for human existence because it prevents thrombotic episodes and spontaneous bleeding [45]. However, NPs may cause an effect, disrupting this balance, when encountering plasma coagulation factors or even cells in circulation [46]. The assessment of blood coagulation pathways (extrinsic, intrinsic, and common) through the PT and APTT is currently accepted as a suitable method to estimate the NP anti- and pro-coagulant properties [43]. Therefore, curcumin loaded glucan NPs and free curcumin were incubated with human plasma for 30 min at 37 °C. Reference values for PT varied in a range of 11 s and 15 s and for APTT between 28 s and 40 s. The results demonstrated that free curcumin at 3.4 µg/mL or encapsulated curcumin at the same concentration (GluCur 100 and GluCur 380) did not show any effect on blood coagulation (Figure 2C). This concentration was chosen because in the hemolysis assay, this concentration of free curcumin was shown to be hemocompatible. PBS was used as a negative control and showed values of PT and APTT of 14.4 s and 33 s, respectively.

**Figure 3:**
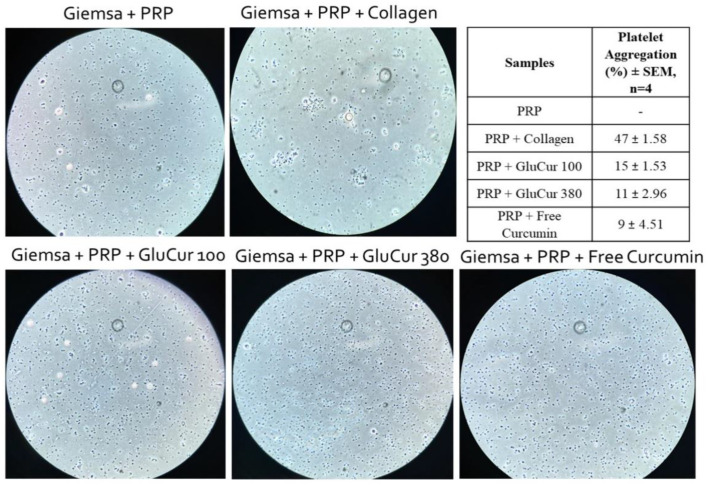
The evaluation of platelet aggregation by incubating platelet-rich plasma (PRP) with curcumin-loaded glucan NPs and free curcumin at 3.4 µg/mL for 30 min. Collagen (600 µg/mL) and PBS were used as the positive and negative controls, respectively. A platelet count was performed and the percentage is outlined in the table (mean ± SEM, n = 4). For the experiment, platelets were stained with Giemsa dye and visualized using an optical microscope.

Platelets are small cells that circulate in the bloodstream and are associated with hemostasis and inflammatory activity [47]. Therefore, these cells are also likely to be in contact with intravenously administered NPs, which can alter platelet function. To elucidate possible interactions between curcumin-loaded glucan NPs or the free curcumin with platelets, platelet-rich plasma was incubated with the different samples at 3.4 µg/mL for 30 min at 37 °C, which were then visualized on a microscope. The platelets stained with Giemsa dye were counted using a Neubauer chamber. The results illustrated in Figure 3 show the presence of stained free platelets in PRP without the presence of aggregates. Nevertheless, when platelets were incubated with collagen (positive control), the formation of platelet aggregates was observed. Thus, at the concentration studied, curcumin loaded NPs and free curcumin were not able to induce platelet aggregation (Figure 3). This result was supported by platelet counting, having obtained percentages of platelet aggregation of 15% (GluCur 100), 11% (GluCur 380), and 9% (free curcumin). According to the literature, a percentage of platelet aggregation above 20% is considered as a positive result [48].

### 3.3. Smaller-Sized Curcumin-Loaded Glucan NPs Report a High Cytotoxic Ability Compared to GluCur 380 NPs in Human PBMCs

**Figure 4:**
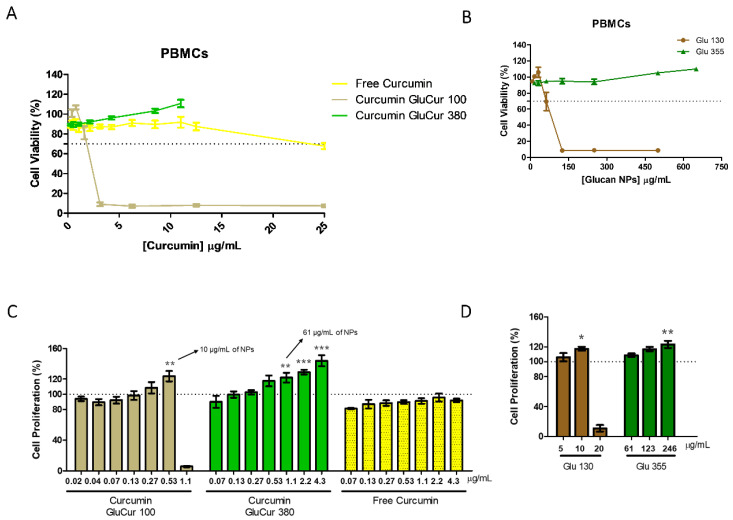
Cytotoxicity effect of curcumin-loaded glucan NPs and free curcumin on human PBMCs. Cell viability was evaluated with the MTT assay after an incubation period of 24 h with increasing concentrations of free drug, GluCur 100, GluCur 380 NPs (**A**), and the unloaded glucan NPs (blank NPs) (**B**) (mean ± SEM, n = 5, five independent experiments, each in triplicate). (**C**) Cell proliferation induced by different concentrations of free curcumin (0.07–4.3 µg/mL) and curcumin associated with GluCur 380 (0.07–4.3 µg/mL) and GluCur 100 NPs (0.02–1.1 µg/mL) after incubation with cells for 72 h and subsequently measured by the MTT assay. Blank NPs were also tested in the same conditions (**D**). Cell culture medium was used as the control (mean ± SEM, n = 4, four independent experiments, each in triplicate). * *p* < 0.05, ** *p* < 0.01, *** *p* < 0.001, when values were significantly different from the control.

NPs as drug delivery systems have been widely used due to their several advantages compared to conventional therapies, namely by improving the bioavailability of the drug, and the drug itself can be targeted to a specific location in the human body, improving its efficacy [49]. However, the nanoparticulate system possesses physicochemical properties that may lead to cell toxicity. In the case of tumoral cells, this cytotoxicity is desirable, however, for healthy cells, it is not, and the effect must be evaluated. Therefore, to assess the cytotoxicity of curcumin-loaded glucan NPs and free curcumin in human PBMCs, the colorimetric MTT assay was used, which measures the metabolic activity of the cells. The results showed that smaller-sized NPs (GluCur 100) were significantly more cytotoxic than larger-sized NPs (GluCur 380). In fact, GluCur 100 NPs led to a decrease in cellular viability for concentrations higher than 2 µg/mL of curcumin encapsulated (corresponding to 40 µg/mL of glucan NP concentration), even reaching only 5% of cell viability for concentrations greater than 4 µg/mL (Figure 4A). The high cytotoxicity of the smaller-sized NPs did not arise from curcumin, because equal concentrations of the free drug were also tested and no cytotoxicity was demonstrated. In contrast, the GluCur 380 NPs did not present cytotoxicity in the concentration range tested (0.27 µg/mL–11 µg/mL of associated curcumin, which corresponds to 15.6 µg/mL–650 µg/mL of NP concentration). The cytotoxic profile of curcumin-unloaded glucan NPs (Glu 130 and Glu 355) was evaluated and it was observed that the higher toxicity of GluCur 100 NPs was solely dependent on the nanoparticle itself (Figure 4B).

When assessing the cellular viability in human PBMCs, both curcumin-loaded glucan NPs at different concentrations exhibited what appeared to be a slight cell stimulation with a cell viability above 100%. To confirm this tendency, a cell proliferation assay was performed using the same colorimetric MTT assay for 72 h. The concentrations of the curcumin-loaded NPs were chosen according to the previous cell viability test. The results presented in Figure 4C showed that free curcumin in the concentration range tested (0.07 µg/mL–4.3 µg/mL) did not induce PBMC proliferation, contradicting the results previously reported by Deters and colleagues [50]. In contrast, GluCur 100 NPs at 0.53 µg/mL of associated curcumin showed an increase in metabolic activity to 124%, which outlines its statistical significance compared to the control (RPMI). This means that cell proliferation was due to the glucan NP itself. In fact, equal concentrations of glucan nanoparticles (10 µg/mL) (Glu 130 NPs without curcumin) were tested and a similar proliferation value (≈120%) was found (Figure 4D). Concentrations of 20 µg/mL of the GluCur 100 NPs containing 1.1 µg/mL curcumin were revealed to be cytotoxic due to the size of the glucan particles (Figure 4C,D). For GluCur 380 NPs, the three highest concentrations tested (1.1 µg/mL, 2.2 µg/mL, and 4.3 µg/mL of associated curcumin, which corresponds to 61 µg/mL, 123 µg/mL, and 246 µg/mL of NP concentration, respectively) stimulated the proliferation of cells to values of 122%, 129%, and 144% of cellular viability. In this case, although free curcumin at the same concentrations was not able to induce cell proliferation, it seems that the synergism between glucan NP and curcumin occurred because Glu 355 NPs (the correspondent NPs without curcumin) only induced significant proliferation at 246 µg/mL (Figure 4C,D).

### 3.4. Both, Curcumin Nanocarriers Reveal a Tendency to Stimulate Human Dendritic Cells to Produce TNF-α

DCs are specialized antigen-presenting cells, responsible for orchestrating innate and adaptive immune responses. For this reason, these cells have been considered as critical players in cancer immunotherapy and novel delivery systems/vaccines are being produced to target DCs [51]. Therefore, it is important to study the interaction of NPs with DCs, namely, their possible ability to induce the secretion of cytokines that help the immune system fight cancer cells. Accordingly, in this work, human Mo-DCs were stimulated with curcumin-loaded glucan nanoparticles for 24 h and the TNF-α, IL-6, IL-12, and IL-10 cytokines were quantified. The different NPs, aside from being produced in LPS-free conditions, the nanocarriers were also previously incubated with 10 µg/mL of polymyxin B sulfate to block the action of any residual endotoxins because of the high sensitivity of the dendritic cells to this type of contamination, and consequently, the appearance of false-positive results.

**Figure 5:**
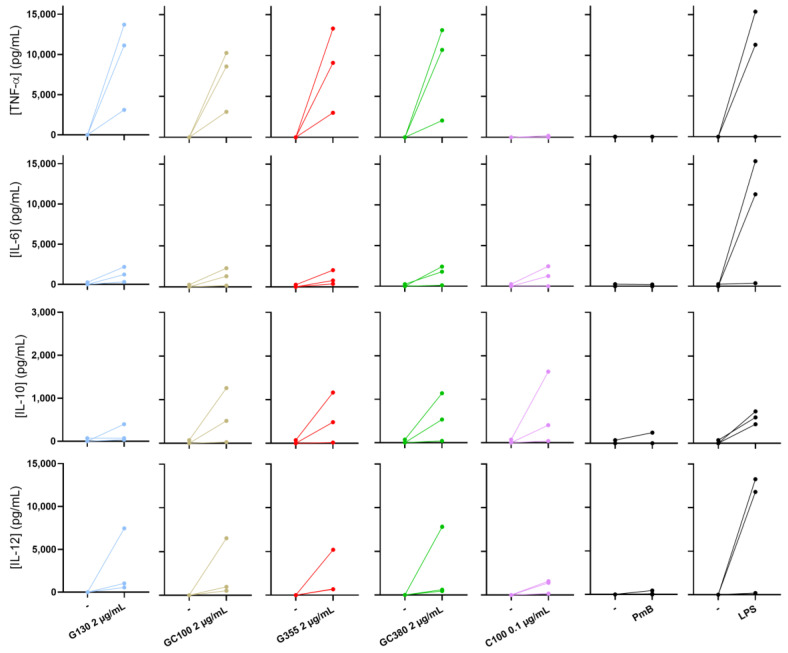
Effects of the curcumin-loaded glucan NPs, blank NPs, and free curcumin in the presence of polymyxin B (PmB), on TNF-α, IL-6, IL-10 and IL-12 secretion by human Mo-DCs, after 24 h of incubation. LPS (20 ng/mL) was used as the positive control. The results display the increase in cytokine secretion compared with the donors’ basal level (–). Data are represented as the mean ± SEM, n = 3.

The data represented in Figure 5 show that all glucan NPs tested induced the production of TNF-α. This is in contrast to the free curcumin, tested in corresponding concentrations to the curcumin encapsulated in GluCur 100 NPs (NPs with the highest curcumin concentration encapsulated), which did not induce this cytokine. These findings demonstrate that the glucan NPs maintain their ability to induce TNF-α production, despite curcumin encapsulation. Moreover, according to the results displayed in Figure 5, both the free drug and all the glucan-based NPs did not seem to have induced the production of IL-6 by a significant amount in any of the donors. Regarding IL-10 and IL-12 secretion, it was observed that the delivery systems were able to induce the production of these cytokines in two of the three donors. The effect of the free curcumin in cells was less noticeable. Notably, the positive control LPS induced a significant TNF-α, IL-6, and IL-12 secretion in two of the three tested donors while PmB did not show any effect, as expected. To avoid misinterpretations regarding the secretion of proinflammatory cytokines in human cells and to communicate the robustness of these results, more donors should be tested in the future. Importantly, none of the tested sample’s concentrations caused cytotoxicity in the dendritic cells during the assay, maintaining cell viabilities greater than 70%, as tested by the Alamar blue assay.

### 3.5. GluCur 380 NPs Decrease Curcumin-Associated Toxicity in a Murine Macrophage Cell Line

**Figure 6:**
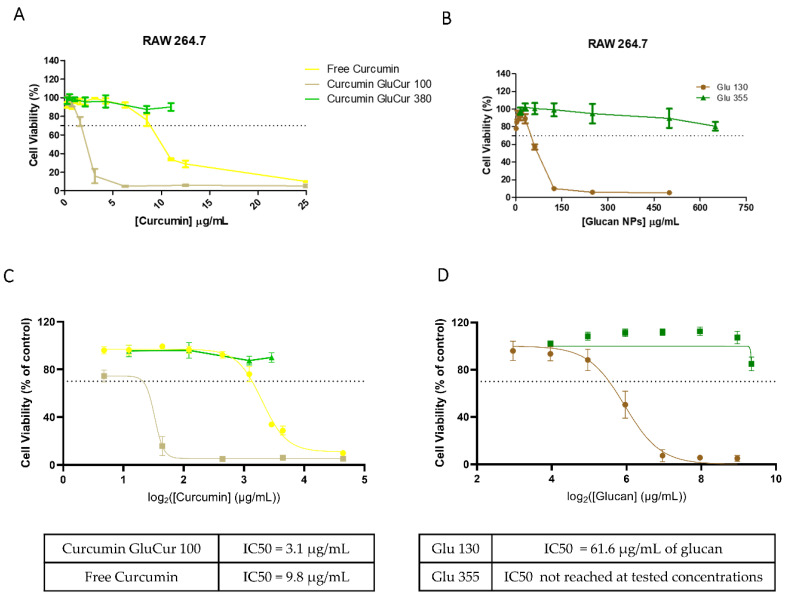
Cytotoxicity effect of the curcumin-loaded glucan NPs and free curcumin on murine RAW 264.7 cells. Cell viability was evaluated with the MTT assay after an incubation period of 24 h with increasing concentrations of free drug, GluCur 100, GluCur 380 NPs (**A**), and the unloaded-curcumin NPs (**B**). Nonlinear regression analysis of the cell viability data, allowing the extrapolation of IC50 values for GluCur 100 and free curcumin (**C**) and for the nanoparticles without curcumin, Glu 130, and Glu 355 (**D**) (mean ± SEM, n = 5, five independent experiments, each in triplicate).

Similarly to the experiments of the PBMC cell viability assessment, the MTT assay was also used to evaluate the toxicity of curcumin-loaded glucan NPs and free curcumin in the RAW 264.7 cells after a 24 h incubation period. The results demonstrated once again that the smaller NPs (GluCur 100 NPs) were more cytotoxic than the larger NPs (Figure 6A). The cytotoxicity profile of free curcumin was also determined, and it was concluded that the toxicity of GluCur 100 was due to the NP and not the encapsulated drug, as concluded in PBMCs (Figure 6B). It was noted that GluCur 100 NPs at 3.1 µg/mL of associated curcumin (62 µg/mL of NP concentration) presented a cellular viability of only 16%, while free curcumin at the same concentration was not toxic (values above the 70% threshold). In contrast to these results, the GluCur 380 NPs were less cytotoxic, and it was possible to see a protective effect on the cells when exposed to curcumin. The cytotoxicity results showed that for concentrations below 11 μg/mL of associated curcumin, the GluCur 380 NPs did not have any effect on the cell viability, contrary to free curcumin, which, at the concentration of 11 μg/mL, presented a decrease in cell viability, reaching 34% (Figure 6A). Figure 6C illustrates a high biocompatibility of the glucan nanoparticles with a size >300 nm. The concentrations tested did not allow us to calculate the IC50 for both nanoparticles, particles with curcumin, GluCur 380 (Figure 6C) or without curcumin, Glu 355 (Figure 6D). Regarding the GluCur 100 NPs, the calculated IC50 was 3.1 μg/mL of curcumin, while for free curcumin, it was 9.8 μg/mL. This high cytotoxicity of the encapsulated curcumin is related to the nanoparticles itself, which is possible to see in Figure 6D. The IC50 of glucan NPs without curcumin (Glu 130) was 61.6 μg/mL.

### 3.6. Curcumin Decreases the Inflammatory Activity of Glucan NPs in RAW 264.7 Cells

Normally, one of the first cell types to make contact with NPs are macrophages. This NP–cell interaction may lead to cytotoxicity, the production of NO, the generation of ROS, or enhance the expression of proinflammatory cytokines, depending on the NP properties [52]. To evaluate the inflammatory or anti-inflammatory activity of curcumin-loaded glucan NPs, the preparation of both NPs was conducted in a sterile and endotoxin-free environment to avoid false-positive results. Moreover, the range of concentrations of the GluCur 100 and GluCur 380 NPs were chosen based on a previous cell viability test to ensure cell viabilities above the 70% threshold, despite having performed the MTT assay (metabolic assay) to confirm the viability after each assay.

**Figure 7:**
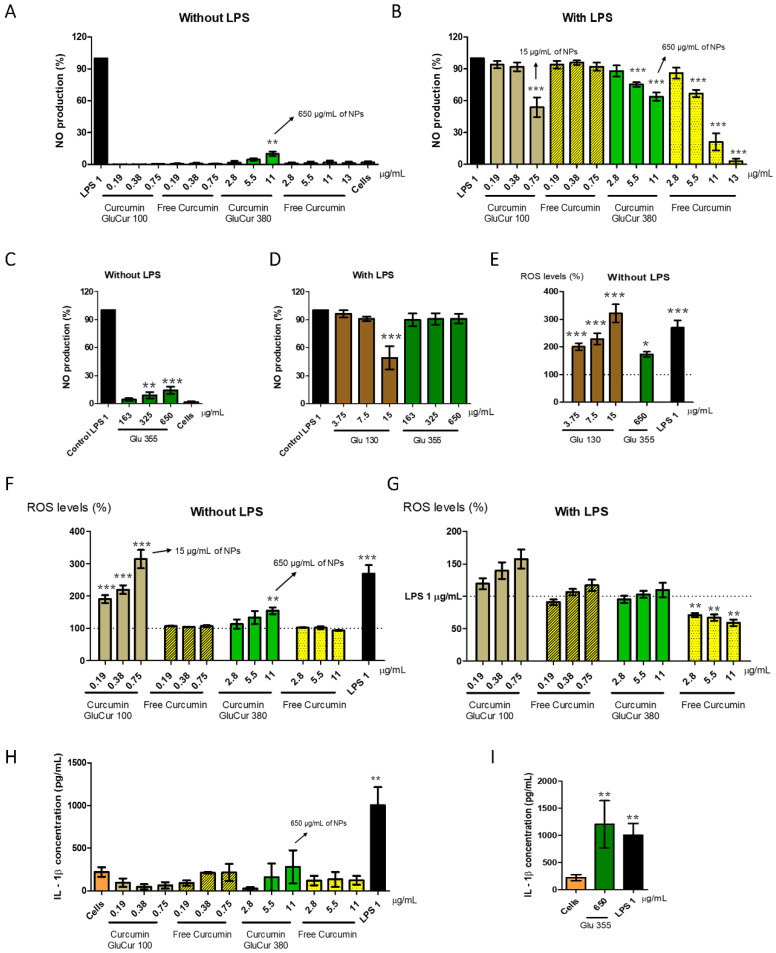
Immunomodulatory effects of curcumin-loaded glucan NPs and the free drug on the RAW 264.7 cell line. (**A**) Assessment of NO production (%) stimulated by endotoxin-free GluCur 100 NPs, GluCur 380 NPs, and the corresponding concentrations of free curcumin as well as blank NPs (Glu 355) (**C**) after 24 h of incubation. LPS (1 μg/mL) and unstimulated cells were used as the positive and negative controls, respectively. (**B**) Inhibition of NO production (%) induced by endotoxin-free GluCur 100 NPs, GluCur 380 NPs, and the corresponding concentrations of free curcumin as well as blank NPs (Glu 130 and Glu 355) (**D**) in the LPS stimulated RAW 264.7 cells after 24 h incubation through the Griess reaction. (**F**) Assessment of ROS production (%) stimulated by free curcumin and curcumin associated with two-sized glucan NPs after 24 h incubation. Additionally, unloaded glucan NPs were also tested in the same conditions (**E**). Samples were carried out in sterile conditions and with endotoxin-free water. LPS (1 μg/mL) and unstimulated cells were used as the positive and negative control, respectively. (**G**) Inhibition of ROS production (%) induced by free curcumin and curcumin associated with glucan NPs after 24 h incubation through the DCFH-DA probe in LPS stimulated RAW 264.7 cells. (**H**) IL-1β secretion levels of the RAW 264.7 cells after being treated with endotoxin-free GluCur 100 NPs, GluCur 380 NPs, and the corresponding concentrations of free curcumin as well as blank NPs (Glu 355) (**I**) for 24 h. This cytokine was quantified by ELISA. Data are presented as the mean ± SEM, n = 4, four independent experiments, each performed in triplicate. * *p* < 0.05, ** *p* < 0.01, and *** *p* < 0.001, when the values were significantly different from the control.

For NO production, the different test samples were incubated with macrophages or with LPS-stimulated macrophages for 24 h, and the NO levels were detected through the Griess reaction. The results showed that GluCur 380 NPs induced a concentration-dependent NO production, being statistically different from the control (cells) at 11 µg/mL of associated curcumin (650 µg/mL of NP concentration) with a 10% production of NO (Figure 7A). However, free curcumin tested in the same range of concentrations did not induce NO production, which means that it was the glucan NP responsible for that effect. The smaller particles (GluCur 100) did not induce the production of NO in the concentrations tested (0.19 µg/mL–0.75 µg/mL of associated curcumin) (Figure 7A). Then, we also investigated the possible anti-inflammatory effects of these particles. It was observed that the GluCur 380 NPs and free curcumin for concentrations equal or greater than 5.5 µg/mL were able to inhibit NO production in a concentration-dependent manner (Figure 7B) and that this effect was more pronounced for free curcumin. This fact was quite clear at the highest concentration tested, where encapsulated curcumin in GluCur 380 NPs presented an inhibition of NO production of 38%, in contrast with the 75% of NO inhibition in the presence of free curcumin (both formulations were significantly different to the positive control, LPS). It seems that because curcumin was encapsulated in the NPs, its effect was attenuated, most probably because curcumin is not readily available and needs to be released from the particle, which, on the other turn, is not a fast process considering the duration of the assay (24 h). An additional concentration of 13 µg/mL of free curcumin was tested to verify if this compound could almost completely suppress the generation of NO, which was demonstrated in Figure 7B with a % of NO production of only 5% (95% inhibition). In parallel to these experiments, the blank NPs were also tested for both NO induction and inhibition. It was confirmed that Glu 355 NPs presented the same stimulatory profile of NO production as the GluCur 380 NPs (Figure 7C). Moreover, we also tested the blank nanoparticles Glu 355, and in the same concentrations of GluCur 380, no decrease in NO production was observed (Figure 7D). Therefore, this observation proved that the effect of inhibition previously observed was due to curcumin and not to the glucan particle itself. Conversely, for GluCur 100 NPs at 0.75 µg/mL of associated curcumin (15 µg/mL of NP concentration), a statistically significant decrease in NO production was observed that was not dependent on curcumin, given that the free drug at the same concentration did not alter the NO levels. Blank NPs of GluCur 100 (Glu 130) were also tested and the same anti-inflammatory effect was observed in the same concentration (Figure 7D). Hence, in the smaller particles, the inhibitory effect of NO was due to the particles.

Concerning ROS production, a cell-permeable fluorescent probe DCFH-DA was used to evaluate the effect of 24 h stimuli of curcumin-loaded glucan NPs in the RAW 264.7 cells and LPS-stimulated RAW 264.7 cells. The results presented in Figure 7E show that Glu 130 NPs, in the same NP concentrations as GluCur 100, induced a high production of ROS as well as Glu 355 NPs in the highest concentration tested (650 µg/mL of the NP concentration, which corresponded to 11 µg/mL of associated curcumin). Furthermore, the data presented in Figure 7F demonstrated that the smaller particles (GluCur 100 NPs) led to the same concentration-dependent ROS generation, and at the highest concentration tested (0.75 µg/mL of associated curcumin which corresponds to 15 µg/mL of NP concentration), the effect was even higher than the positive control LPS with the % of ROS production of 315% and 269%, respectively. Free curcumin tested in the same range of concentrations as GluCur 100 NPs did not stimulate the production of ROS, evidence that the production of ROS was due to the delivery system and not the drug. Similarly, GluCur 380 NPs also demonstrated a tendency to produce ROS, depending on the concentration. However, the only significant increase of ROS was at 11 µg/mL of associated curcumin (650 µg/mL of NP concentration) with ROS levels of 155%. Free curcumin ranging from 2.8 µg/mL to 11 µg/mL did not lead to the generation of ROS. Nevertheless, despite the inflammatory activity verified by both curcumin-loaded glucan NPs, the results presented in Figure 7G show the potential of curcumin (in concentrations equal to or higher than 2.8 µg/mL) to inhibit the LPS stimulated ROS production in a statistically significant manner. This decrease in ROS levels was concentration-dependent with ROS levels of 71%, 67%, and 59%, respectively.

Regarding the assessment of proinflammatory cytokines, the supernatants of the RAW 264.7 cells were tested to determine the ability of curcumin-loaded glucan NPs to secrete IL-1β, a cytokine considered crucial for the regulation of the inflammatory response [53]. As observed in Figure 7H, LPS (1 µg/mL) induced adequate secretion levels of IL-1β (around 1004 pg/mL), while both curcumin nanocarriers and the free drug did not lead to the secretion of IL-1β. Blank NPs (Glu 130 and Glu 355) were also tested, and an interesting result was detected, where the larger particles at 650 µg/mL were able to significantly increase the secretion of this cytokine (Figure 7I). Nevertheless, the same delivery system, but with curcumin encapsulated (GluCur 380) at the same NP concentration (which corresponds to 11 µg/mL of associated curcumin) did not present IL-1β secretion, clearly showing the anti-inflammatory effect of curcumin.

## 4. Discussion

Despite curcumin presenting several biological properties [54], its application as a potential drug has still not been approved due to its hydrophobicity and low bioavailability [55]. Therefore, to surpass these problems, the main focus of the work presented here was to develop a preparation method to obtain two glucan nanoparticles with different sizes that were capable of encapsulating the curcumin with high loading efficacy, and study the influence of the size on different biological parameters. For this, polymeric nanoparticles were obtained by a nanoprecipitation method and curdlan was the polymer selected. β-Glucans are particularly interesting candidates for delivery systems not only due to their biocompatibility, but also because glucans can be recognized by pattern recognition receptors of immune cells, specifically dectin-1, which means that this polymer can be efficiently taken up by such cells, offering a targeted drug delivery system with immunomodulatory ability [56].

In this work, we tested two NPs with different sizes because it was observed by our group in previous studies that different sized-glucan NPs caused a different immunotoxicity profile [38]. Therefore, we encapsulated curcumin in NPs with similar sizes as the ones previously tested.

The results herein presented revealed that by manipulating the polymer’s concentration, two different-sized curcumin-loaded glucan NPs (GluCur 100 and GluCur 380) were produced, both highly effective in encapsulating curcumin.

Before testing the efficacy of these curcumin nanoparticles, it is of utmost importance to test their immunotoxicity to ensure their safety [57]. This approach is being increasingly accepted by the scientific community when the intention is to bring the product to the nanomedicine market [58].

Regarding the hemocompatibility assessment, our study revealed that although glucan is a natural, non-toxic polymer, nanoparticles made of this material presented hemolytic activity, with the smaller particles being the most toxic ones. This finding is in accordance with a previous report from our group that highlights the importance of not assuming that the NPs would behave like the bulk polymer [34]. Moreover, the fact that smaller curcumin-loaded particles are more toxic to erythrocytes is not surprising because due to their smaller size, they can more easily penetrate the cells, causing cell death, while the bigger particles may enter through a different mechanism. Considering the encapsulated drug, our study demonstrated that both curcumin-loaded glucan nanoparticles were more hemolytic than the free drug. This result is expected, taking into account Deka’s study, which had already established a non-hemolytic effect of free curcumin with goat blood at concentrations higher than 250 µg/mL [59]. With regard to the thrombogenic properties, we found that the GluCur 100 and GluCur 380 NPs had no effect on the extrinsic and intrinsic coagulation pathways (PT and APTT, respectively). In a previous study, Huang and colleagues reported the preparation of β-glucan-blending polyvinyl alcohol films, where the content of glucan varied from 10% to 60% and in the highest % of this polymer, we detected no alteration in the coagulation time [60]. Furthermore, our results show that curcumin also did not affect the coagulation cascade, however, these results are contradictory to the literature, where curcumin was shown to have a potent anticoagulant activity by prolonging APTT in a concentration-dependent manner [61,62]. In our study, at the concentration of 3.4 μg/mL (free curcumin), the APTT had a value of 33.3 s, while in the paper by Dong-Chan Kim and colleagues, APTT in a similar concentration had a value of 52.6 s [61]. However, their experimental conditions were unlike ours, as they used an incubation time of human plasma with curcumin of 1 min, while we used 30 min, which can explain the differences. Finally, it was observed that curcumin-loaded glucan nanoparticles and free curcumin did not induce platelet aggregation. These results are quite interesting because in a previous study by our group, the glucan nanoparticles (Glu 130 and Glu 355) alone induced platelet aggregation [38]. Consequently, our study revealed that curcumin can inhibit glucan-induced platelet aggregation. Accordingly, previous studies have also reported that curcumin inhibited platelet aggregation induced by several agonists such as arachidonic acid (AA), collagen, adenosine diphosphate (ADP), and thrombin [63,64,65]. This outcome was explained by the fact that curcumin can act as a cyclooxygenase inhibitor and hamper thromboxane A2 formation, which blocks the glycoprotein IIb/IIIa receptor, inhibiting platelet aggregation [66].

In this paper, we proposed two new nanomedicines that can target the immune system, and can therefore potentially alter human health homeostasis. To assess the toxicity of the materials, we selected a primary culture and a cell line: human PBMCs as illustrative of various types of immune cells and murine macrophages (RAW 264.7 cells). As the literature suggests, one of the objectives to encapsulate bioactive compounds is to reduce the cytotoxicity of the compound. However, depending on the cytotoxicity of the nanocarrier (in our case GluCur 100 or GluCur 380), the protective effect of the encapsulation of curcumin can be noticeable or not. Mai Huong Le and colleagues demonstrated that curcumin-loaded glucan nanoparticles (glucan was isolated from a mushroom) were more toxic than free curcumin in both the Hep-G2 and LU-1 cell lines [67], which is similar to what happened with our GluCur 100 NPs in the PBMCs and RAW 264.7 cells. The authors argued that the increase in the solubility of curcumin and compatibility between glucan and curcumin led to the effectiveness in the activity of this compound. Another study by Zulaikha Busari and co-workers revealed a similar result, and the curcumin-loaded PLGA nanoparticles had higher toxicity compared with free curcumin in the RAW 264.7 cells [68]. They reported that this was due to the better absorption of the NPs influenced by their smaller size, although they did not evaluate blank particles of the same size, which could have confirmed their hypothesis. On the other hand, a study with results similar to that of the GluCur 380 NPs was reported by Jiao Wang and colleagues, where they showed that curcumin encapsulated in solid lipid nanoparticles (SLNs) was less toxic than free curcumin in the HEK293T and LO2 cells [69], as was our case in the RAW 264.7 cells. Interestingly, in human PBMCs, this result was not replicated because free curcumin was less toxic than in the RAW 264.7 cells. We can hypothesize that the uptake by monocytes and lymphocytes of curcumin, when not associated with glucan as a nanocarrier, is more reduced compared to macrophages that possess a great phagocytic ability [70]. The cytotoxicity of free curcumin has been described by two groups in PBMCs who reported its cytocompatibility at least up to 25 µg/mL, consistent with our study [71,72]. Considering the RAW 264.7 cell viability, two other groups reported superior viability of free curcumin compared to what we obtained [59,68]. This difference between the reports is likely because despite using the same cell line, the cellular concentration, the cell passage, the time of incubation, or even the solvent used to dissolve curcumin may be different, which would lead to different outcomes.

Macrophages are antigen-presenting cells with a critical function in our organism. When activated, these cells can generate nitrogen and reactive oxygen species as well as secrete a variety of proinflammatory cytokines that are capable of killing cancer cells or other pathogens [73]. Concerning the ability to produce NO by macrophages, the literature review suggested that free curcumin has an inhibitory effect in NO production. One of the studies revealed a concentration-dependent decrease in NO generation for concentrations above 2 µg/mL of curcumin in LPS-stimulated RAW 264.7 cells after 24 h incubation due to the inhibition of inducible NO synthase activity [74]. In our report, the same inhibitory effect for similar concentrations of free curcumin was detected, confirming the results of previous studies. The larger particles with concentrations greater than 5 µg/mL of curcumin encapsulated maintained the ability to inhibit NO production. This effect was also observed in the paper by Liu et al., in which they produced curcumin-loaded rice bran albumin NPs with concentrations of encapsulation similar to ours, the same inhibitory profile of NO production in the particles and in the free drug was seen [75]. On the other hand, curcumin-loaded GluCur 380 NPs were able to induce the production of NO in macrophages due to the glucan particle itself. Regarding the literature, it is suggested that the activation of macrophages leading to the production of the inflammatory mediator NO is dependent on the chemical structure of the glucans. Li and colleagues showed a slight production of NO in macrophages with the same polymer as the one we used to produce the particles in similar concentrations, which is in agreement with our results [76]. Moreover, previous results from our group had already revealed the potential of the curdlan delivery system (Glu 355 NPs) to induce the production of NO in murine macrophages [38]. Regarding proinflammatory cytokine induction, activated macrophages induce the release of interleukin-1β (IL-1β), which is crucial for host defense responses against pathogens [77]. Moreover, it has been reported that the size of β-glucans differentially affects this cytokine secretion [78]. In the literature, recent reports have outlined the inhibitory effect of curcumin on IL-1β production [79,80]. Some authors argue that the decreased expression of inflammatory cytokines such as IL-1β and even TNF-α results from the inhibition of the transcription factor NF-kB and consequent suppression of the NLRP3 inflammasome [69]. This effect was confirmed by our results, which showed the inability of curcumin to induce cells to produce IL-1β as well as the efficiency of this compound to inhibit the production of IL-1β in encapsulated curcumin glucan NPs (GluCur 380).

Dendritic cells are key candidates in tumor immunotherapy due to the properties of their natural adjuvants. For this reason, in recent years, one of the crucial strategies that is gradually being implemented in the field of vaccine design is the production of delivery systems that can target DCs, namely, by using polymers such as curdlan [81]. Accordingly, the effect of curdlan on dendritic cells has been well-described in the literature [82,83,84]. It is known that curdlan is an agonist of dectin-1 and Toll-like receptor 2/6, which are receptors present in dendritic cells (and other immune cells), and their activation can trigger molecular pathways that induce a wide array of immune responses such as increasing the expression levels of certain cytokines or even promoting the induction of antitumor Th9 cells [85]. Kim and colleagues reported the stimulation of TNF-α and IL-12 was induced by 10 µg/mL of curdlan in the bone marrow-derived immature DCs using the same incubation period as our data. The authors concluded that curdlan is a good stimulator of DCs and hence a useful adjuvant for cancer immunotherapy [86]. Furthermore, Elder and coworkers described the stimulation of two-sized particulate β-glucans in Mo-DCs and they demonstrated that both particles induced proinflammatory cytokines, but the smaller ones were induced to a lesser extent [78]. This appears discordant with our results, but it can be explained by the minor difference in the sizes of our delivery systems. Curcumin, on the other hand, did not trigger the induction of cytokine production by these cells. Therefore, our results reveal that curdlan retained its TNF-α inducing activity in human Mo-DCs while nanoparticulated, and that this property was not affected by the presence of curcumin in the particles.

Cancer cells exhibit greater levels of ROS compared with normal cells not only because of their hypermetabolism, but also because these cells present a reduced antioxidant capacity [87]. Therefore, ROS-generating formulations could more easily target cancer cells, promoting a redox imbalance, eventually leading to cell death. Consequently, several prooxidative agents have been studied as anticancer drugs, namely, glucan. The antitumor activity of this polysaccharide has been attributed to its capacity to be recognized by different immune receptors, fostering their activation and helping the host to constitute a strong immune response by stimulating cell apoptosis through the ROS stress response pathway [88]. Regarding ROS production, our study revealed the great potential of both curcumin-loaded glucan NPs to stimulate ROS cellular generation, particularly, the smaller particles in murine macrophages. A similar result has already been published in which a particulate beta-glucan induced a significant amount of ROS (concentration-dependent) after exposure in J774A.1 (mouse macrophage cell line) [89]. Furthermore, Jia and colleagues also reported the construction of selenium nanoparticle/β-glucan composites in which the stimulating effect of ROS production was dependent on the size of the particles, where smaller sizes led to higher levels of ROS [90], as reported in our study. However, despite several articles revealing the anticarcinogenic effect of curcumin, its mechanism to achieve cancer cell death is different from glucan: depending on the concentration of curcumin, it can act as an antioxidant or pro-oxidant agent [91]. It then becomes important to make sure that the encapsulated curcumin is not masking the desired effect caused by the nanocarrier glucan. From our report, we concluded that in the range of concentrations tested, the curcumin-loaded glucan NPs maintained the same profile as the unloaded delivery system, which means that curcumin and glucan are compatible to act as an improved system against cancer. Moreover, free curcumin up to 11 µg/mL did not present pro-oxidant activity by itself, which is in accordance with the literature that reports that this agent can only induce ROS generation with concentrations higher than 20 µg/mL [92].

From a general point of view, this paper revealed the potential of GluCur 100 NPs for use as a chemotherapeutic agent against cancer due to their high cytotoxicity, although more studies are needed in the future to ensure that intratumoral administration would be the safest route to avoid any type of adverse effect. Conversely, GluCur 380 NPs showed a greater capacity to be used as an immunotherapeutic agent on account of its ability to activate immune cells such as Mo-DCs and macrophages, leading to proinflammatory cytokine production as well as ROS generation, independently of cell death.

## 5. Conclusions

Over the past decade, curcumin has been suggested as a potential therapeutic agent in various inflammation-based diseases. Nevertheless, its clinical application is limited due to poor bioavailability. Several carriers to encapsulate curcumin have already been produced and explored to improve its anticancer efficacy, however, a considerable number of papers lacked the assessment of immunotoxicity data and proper NP characterization. The work presented here intended to address this issue to anticipate possible toxicity concerns in the initial phase of product development. Two differently sized curdlan delivery systems were manufactured as a nanocarrier for curcumin and studied in terms of their size, surface properties, and morphology. Our paper revealed that curcumin-loaded glucan NPs four times the size of 100 nm created an immunotoxicity profile characterized by proinflammatory and pro-oxidant properties without being associated with cellular toxicity, unlike the smaller particles. Therefore, the immunotoxicity shown was dependent on the precise size of the glucan nanoparticles. For this reason, the weight-of-evidence decision-making approach for immunotoxicity testing for new nanomedicines should be carefully considered since the same polymer, in this case, glucan, the raw material, or nanoparticles with different sizes may have substantially different immunological properties.

To conclude, more information such as this report would contribute to an increase in cancer nanomedicine safety and success during their introduction in the market.

## Data Availability

Not applicable.

## References

[1] Ursini F., Maiorino M., Forman H.J. (2016). Redox homeostasis: The Golden Mean of healthy living. Redox Biol..

[2] Gorrini C., Harris I.S., Mak T.W. (2013). Modulation of oxidative stress as an anticancer strategy. Nat. Rev. Drug Discov..

[3] Joseph A., Wood T., Chen C.-C., Corry K., Snyder J.M., Juul S.E., Parikh P., Nance E. (2018). Curcumin-loaded polymeric nanoparticles for neuroprotection in neonatal rats with hypoxic-ischemic encephalopathy. Nano Res..

[4] Sampath M., Lakra R., Korrapati P., Sengottuvelan B. (2014). Curcumin loaded poly (lactic-co-glycolic) acid nanofiber for the treatment of carcinoma. Colloids Surf. B Biointerfaces.

[5] Jurenka J.S. (2009). Anti-inflammatory properties of curcumin, a major constituent of Curcuma longa: A review of preclinical and clinical research. Altern. Med. Rev..

[6] Salehi B., Stojanović-Radić Z., Matejić J., Sharifi-Rad M., Anil Kumar N.V., Martins N., Sharifi-Rad J. (2019). The therapeutic potential of curcumin: A review of clinical trials. Eur. J. Med. Chem..

[7] Rai M., Pandit R., Gaikwad S., Yadav A., Gade A. (2015). Potential applications of curcumin and curcumin nanoparticles: From traditional therapeutics to modern nanomedicine. Nanotechnol. Rev..

[8] Abu-Taweel G.M., Attia M.F., Hussein J., Mekawi E.M., Galal H.M., Ahmed E.I., Allam A.A., El-Naggar M.E. (2020). Curcumin nanoparticles have potential antioxidant effect and restore tetrahydrobiopterin levels in experimental diabetes. Biomed. Pharmacother..

[9] Gómez-Estaca J., Balaguer M.P., López-Carballo G., Gavara R., Hernández-Muñoz P. (2017). Improving antioxidant and antimicrobial properties of curcumin by means of encapsulation in gelatin through electrohydrodynamic atomization. Food Hydrocoll..

[10] Wang X., Huang H., Chu X., Han Y., Li M., Li G., Liu X. (2019). Encapsulation and binding properties of curcumin in zein particles stabilized by Tween 20. Colloids Surf. A Physicochem. Eng. Asp..

[11] Wojcik M., Krawczyk M., Wojcik P., Cypryk K., Wozniak L.A. (2018). Molecular Mechanisms Underlying Curcumin-Mediated Therapeutic Effects in Type 2 Diabetes and Cancer. Oxidative Med. Cell. Longev..

[12] Aggeli I.-K., Koustas E., Gaitanaki C., Beis I. (2013). Curcumin Acts as a Pro-Oxidant Inducing Apoptosis Via JNKs in the Isolated PerfusedRana ridibundaHeart. J. Exp. Zool. Part A Ecol. Genet. Physiol..

[13] López-Lázaro M. (2008). Anticancer and carcinogenic properties of curcumin: Considerations for its clinical development as a cancer chemopreventive and chemotherapeutic agent. Mol. Nutr. Food Res..

[14] Li P.-M., Li Y.-L., Liu B., Wang W.-J., Wang Y.-Z., Li Z. (2014). Curcumin Inhibits MHCC97H Liver Cancer Cells by Activating ROS/TLR-4/Caspase Signaling Pathway. Asian Pac. J. Cancer Prev..

[15] Talib W.H., Al-hadid S.A., Wild Ali M.B., Al-Yasari I.H., Abd Ali M.R. (2018). Role of curcumin in regulating p53 in breast cancer: An overview of the mechanism of action. Breast Cancer Targets Ther..

[16] Catanzaro M., Corsini E., Rosini M., Racchi M., Lanni C. (2018). Immunomodulators Inspired by Nature: A Review on Curcumin and Echinacea. Molecules.

[17] Hewlings S., Kalman D. (2017). Curcumin: A Review of Its Effects on Human Health. Foods.

[18] Basnet P., Skalko-Basnet N. (2011). Curcumin: An Anti-Inflammatory Molecule from a Curry Spice on the Path to Cancer Treatment. Molecules.

[19] Dei Cas M., Ghidoni R. (2019). Dietary Curcumin: Correlation between Bioavailability and Health Potential. Nutrients.

[20] Kharat M., Du Z., Zhang G., McClements D.J. (2017). Physical and Chemical Stability of Curcumin in Aqueous Solutions and Emulsions: Impact of pH, Temperature, and Molecular Environment. J. Agric. Food Chem..

[21] Anand P., Kunnumakkara A.B., Newman R.A., Aggarwal B.B. (2007). Bioavailability of Curcumin: Problems and Promises. Mol. Pharm..

[22] Huong L.M., Thu H.P., Thuy N.T.B., Ha T.T.H., Thi H.T.M., Trang M.T., Hang T.T.N., Nghi D.H., Phuc N.X., Quang D.T. (2011). Preparation and Antitumor-promoting Activity of Curcumin Encapsulated by 1,3-β-Glucan Isolated from Vietnam Medicinal MushroomHericium erinaceum. Chem. Lett..

[23] Wang K., Zhang T., Liu L., Wang X., Wu P., Chen Z., Ni C., Zhang J., Hu F., Huang J. (2012). Novel micelle formulation of curcumin for enhancing antitumor activity and inhibiting colorectal cancer stem cells. Int. J. Nanomed..

[24] Li L., Braiteh F.S., Kurzrock R. (2005). Liposome-encapsulated curcumin. Cancer.

[25] Rafati N., Zarrabi A., Caldera F., Trotta F., Ghias N. (2019). Pyromellitic dianhydride crosslinked cyclodextrin nanosponges for curcumin controlled release; formulation, physicochemical characterization and cytotoxicity investigations. J. Microencapsul..

[26] Casula L., Lai F., Pini E., Valenti D., Sinico C., Cardia M.C., Marceddu S., Ailuno G., Fadda A.M. (2021). Pulmonary Delivery of Curcumin and Beclomethasone Dipropionate in a Multicomponent Nanosuspension for the Treatment of Bronchial Asthma. Pharmaceutics.

[27] Zhang J., Tang Q., Xu X., Li N. (2013). Development and evaluation of a novel phytosome-loaded chitosan microsphere system for curcumin delivery. Int. J. Pharm..

[28] Pawar H., Wankhade S.R., Yadav D.K., Suresh S. (2015). Development and evaluation of co-formulated docetaxel and curcumin biodegradable nanoparticles for parenteral administration. Pharm. Dev. Technol..

[29] Kaur R., Sharma M., Ji D., Xu M., Agyei D. (2019). Structural Features, Modification, and Functionalities of Beta-Glucan. Fibers.

[30] Han B., Baruah K., Cox E., Vanrompay D., Bossier P. (2020). Structure-Functional Activity Relationship of β-Glucans From the Perspective of Immunomodulation: A Mini-Review. Front. Immunol..

[31] Zhou J.-L., Song F., Tian J.-F., Nie W.-C., Wang X.-L., Wang Y.-Z. (2017). Electrostatic wrapping of doxorubicin with curdlan to construct an efficient pH-responsive drug delivery system. Nanotechnology.

[32] Jin Y., Li P., Wang F. (2018). β-glucans as potential immunoadjuvants: A review on the adjuvanticity, structure-activity relationship and receptor recognition properties. Vaccine.

[33] Schepetkin I.A., Quinn M.T. (2006). Botanical polysaccharides: Macrophage immunomodulation and therapeutic potential. Int. Immunopharmacol..

[34] Jesus S., Marques A.P., Duarte A., Soares E., Costa J.P., Colaço M., Schmutz M., Som C., Borchard G., Wick P. (2020). Chitosan Nanoparticles: Shedding Light on Immunotoxicity and Hemocompatibility. Front. Bioeng. Biotechnol..

[35] Foroozandeh P., Aziz A.A. (2018). Insight into Cellular Uptake and Intracellular Trafficking of Nanoparticles. Nanoscale Res. Lett..

[36] Varlamova E.G., Gudkov S.V., Plotnikov E.Y., Turovsky E.A. (2022). Size-Dependent Cytoprotective Effects of Selenium Nanoparticles during Oxygen-Glucose Deprivation in Brain Cortical Cells. Int. J. Mol. Sci..

[37] Shang L., Nienhaus K., Nienhaus G.U. (2014). Engineered nanoparticles interacting with cells: Size matters. J. Nanobiotechnol..

[38] Colaço M., Marques A.P., Jesus S., Duarte A., Borges O. (2020). Safe-by-Design of Glucan Nanoparticles: Size Matters When Assessing the Immunotoxicity. Chem. Res. Toxicol..

[39] Nair A.V., Gummadi S.N., Doble M. (2016). Characterization and biological activities of cyclic (1 → 3, 1 → 6)-β-glucans from Bradyrhizobium japonicum. Biotechnol. Lett..

[40] Halamoda-Kenzaoui B., Bremer-Hoffmann S. (2018). Main trends of immune effects triggered by nanomedicines in preclinical studies. Int. J. Nanomed..

[41] Schneider C., Gordon O.N., Edwards R.L., Luis P.B. (2015). Degradation of Curcumin: From Mechanism to Biological Implications. J. Agric. Food Chem..

[42] Hannon G., Lysaght J., Liptrott N.J., Prina-Mello A. (2019). Immunotoxicity Considerations for Next Generation Cancer Nanomedicines. Adv. Sci..

[43] Dobrovolskaia M.A., McNeil S.E. (2013). Understanding the correlation between in vitro and in vivo immunotoxicity tests for nanomedicines. J. Control. Release.

[44] (2013). Standard Test Method for Analysis of Hemolytic Properties of Nanoparticles.

[45] Brenner B., Lisman T. (2018). Hemostasis and Thrombosis in Extreme Physiological and Pathological Conditions. Semin. Thromb. Hemost..

[46] Dobrovolskaia M.A. (2015). Pre-clinical immunotoxicity studies of nanotechnology-formulated drugs: Challenges, considerations and strategy. J. Control. Release.

[47] Jenne C.N., Kubes P. (2015). Platelets in inflammation and infection. Platelets.

[48] Tsoupras A., Zabetakis I., Lordan R. (2019). Platelet aggregometry assay for evaluating the effects of platelet agonists and antiplatelet compounds on platelet function in vitro. MethodsX.

[49] Jain A.K., Thareja S. (2019). In vitro and in vivo characterization of pharmaceutical nanocarriers used for drug delivery. Artif. Cells Nanomed. Biotechnol..

[50] Deters M., Knochenwefel H., Lindhorst D., Koal T., Meyer H.H., Hänsel W., Resch K., Kaever V. (2008). Different Curcuminoids Inhibit T-Lymphocyte Proliferation Independently of Their Radical Scavenging Activities. Pharm. Res..

[51] Bol K.F., Schreibelt G., Rabold K., Wculek S.K., Schwarze J.K., Dzionek A., Teijeira A., Kandalaft L.E., Romero P., Coukos G. (2019). The clinical application of cancer immunotherapy based on naturally circulating dendritic cells. J. ImmunoTherapy Cancer.

[52] Rothen-Rutishauser B., Bourquin J., Petri-Fink A. (2019). Nanoparticle-Cell Interactions: Overview of Uptake, Intracellular Fate and Induction of Cell Responses. Biological Responses to Nanoscale Particles.

[53] Martín-Sánchez F., Diamond C., Zeitler M., Gomez A.I., Baroja-Mazo A., Bagnall J., Spiller D., White M., Daniels M.J.D., Mortellaro A. (2016). Inflammasome-dependent IL-1β release depends upon membrane permeabilisation. Cell Death Differ..

[54] Tsuda T. (2018). Curcumin as a functional food-derived factor: Degradation products, metabolites, bioactivity, and future perspectives. Food Funct..

[55] Mirzaei H., Shakeri A., Rashidi B., Jalili A., Banikazemi Z., Sahebkar A. (2017). Phytosomal curcumin: A review of pharmacokinetic, experimental and clinical studies. Biomed. Pharmacother..

[56] Sun Y., Duan B., Chen H., Xu X. (2020). A Novel Strategy for Treating Inflammatory Bowel Disease by Targeting Delivery of Methotrexate through Glucan Particles. Adv. Healthc. Mater..

[57] Lopalco A., Denora N., Nicolotti O. (2018). Nanoformulations for Drug Delivery: Safety, Toxicity, and Efficacy. Computational Toxicology Methods in Molecular Biology.

[58] Kraegeloh A., Suarez-Merino B., Sluijters T., Micheletti C. (2018). Implementation of Safe-by-Design for Nanomaterial Development and Safe Innovation: Why We Need a Comprehensive Approach. Nanomaterials.

[59] Deka C., Aidew L., Devi N., Buragohain A.K., Kakati D.K. (2016). Synthesis of curcumin-loaded chitosan phosphate nanoparticle and study of its cytotoxicity and antimicrobial activity. J. Biomater. Sci. Polym. Ed..

[60] Huang M.-H., Yang M.-C. (2008). Evaluation of glucan/poly(vinyl alcohol) blend wound dressing using rat models. Int. J. Pharm..

[61] Kim D.-C., Ku S.-K., Bae J.-S. (2012). Anticoagulant activities of curcumin and its derivative. BMB Rep..

[62] Duse L., Agel M.R., Pinnapireddy S.R., Schäfer J., Selo M.A., Ehrhardt C., Bakowsky U. (2019). Photodynamic Therapy of Ovarian Carcinoma Cells with Curcumin-Loaded Biodegradable Polymeric Nanoparticles. Pharmaceutics.

[63] Keihanian F., Saeidinia A., Bagheri R.K., Johnston T.P., Sahebkar A. (2018). Curcumin, hemostasis, thrombosis, and coagulation. J. Cell. Physiol..

[64] Perrone D., Ardito F., Giannatempo G., Dioguardi M., Troiano G., Lo Russo L., De Lillo A., Laino L., Lo Muzio L. (2015). Biological and therapeutic activities, and anticancer properties of curcumin. Exp. Ther. Med..

[65] Ngo T., Kim K., Bian Y., An G.-J., Bae O.-N., Lim K.-M., Chung J.-H. (2019). Cyclocurcumin from Curcuma longa selectively inhibits shear stress-induced platelet aggregation. J. Funct. Foods.

[66] Tabeshpour J., Hashemzaei M., Sahebkar A. (2018). The regulatory role of curcumin on platelet functions. J. Cell. Biochem..

[67] Le M.H., Do H.D., Tran Thi H.H., Dung L.V., Nguyen H.N., Tran Thi H.N., Nguyen L.D., Hoang C.K., Le H.C., Le Thi T.H. (2016). The dual effect of curcumin nanoparticles encapsulated by 1-3/1-6 β-glucan from medicinal mushrooms Hericium erinaceus and Ganoderma lucidum. Adv. Nat. Sci. Nanosci. Nanotechnol..

[68] Busari Z.A., Dauda K.A., Morenikeji O.A., Afolayan F., Oyeyemi O.T., Meena J., Sahu D., Panda A.K. (2017). Antiplasmodial Activity and Toxicological Assessment of Curcumin PLGA-Encapsulated Nanoparticles. Front. Pharmacol..

[69] Wang J., Zhu R., Sun D., Sun X., Geng Z., Liu H., Wang S.-L. (2015). Intracellular Uptake of Curcumin-Loaded Solid Lipid Nanoparticles Exhibit Anti-Inflammatory Activities Superior to Those of Curcumin Through the NF-κB Signaling Pathway. J. Biomed. Nanotechnol..

[70] Hirayama D., Lida T., Nakase H. (2017). The Phagocytic Function of Macrophage-Enforcing Innate Immunity and Tissue Homeostasis. Int. J. Mol. Sci..

[71] Khatun B., Banik N., Hussain A., Ramteke A., Maji T. (2018). Genipin crosslinked curcumin loaded chitosan/montmorillonite K-10 (MMT) nanoparticles for controlled drug delivery applications. J. Microencapsul..

[72] Singh A., Lavkush, Kureel A.K., Dutta P.K., Kumar S., Rai A.K. (2018). Curcumin loaded chitin-glucan quercetin conjugate: Synthesis, characterization, antioxidant, in vitro release study, and anticancer activity. Int. J. Biol. Macromol..

[73] Nishanth R.P., Jyotsna R.G., Schlager J.J., Hussain S.M., Reddanna P. (2011). Inflammatory responses of RAW 264.7 macrophages upon exposure to nanoparticles: Role of ROS-NFκB signaling pathway. Nanotoxicology.

[74] Ben P., Liu J., Lu C., Xu Y., Xin Y., Fu J., Huang H., Zhang Z., Gao Y., Luo L. (2011). Curcumin promotes degradation of inducible nitric oxide synthase and suppresses its enzyme activity in RAW 264.7 cells. Int. Immunopharmacol..

[75] Liu C., Yang X., Wu W., Long Z., Xiao H., Luo F., Shen Y., Lin Q. (2018). Elaboration of curcumin-loaded rice bran albumin nanoparticles formulation with increased in vitro bioactivity and in vivo bioavailability. Food Hydrocoll..

[76] Li P., Zhang X., Cheng Y., Li J., Xiao Y., Zhang Q., Zong A., Zhong C., Wang F. (2014). Preparation and in vitro immunomodulatory effect of curdlan sulfate. Carbohydr. Polym..

[77] Lopez-Castejon G., Brough D. (2011). Understanding the mechanism of IL-1β secretion. Cytokine Growth Factor Rev..

[78] Elder M.J., Webster S.J., Chee R., Williams D.L., Hill Gaston J.S., Goodall J.C. (2017). β-Glucan Size Controls Dectin-1-Mediated Immune Responses in Human Dendritic Cells by Regulating IL-1β Production. Front. Immunol..

[79] Yin H., Guo Q., Li X., Tang T., Li C., Wang H., Sun Y., Feng Q., Ma C., Gao C. (2018). Curcumin Suppresses IL-1β Secretion and Prevents Inflammation through Inhibition of the NLRP3 Inflammasome. J. Immunol..

[80] Guglielmo A., Sabra A., Elbery M., Cerveira M.M., Ghenov F., Sunasee R., Ckless K. (2017). A mechanistic insight into curcumin modulation of the IL-1β secretion and NLRP3 S-glutathionylation induced by needle-like cationic cellulose nanocrystals in myeloid cells. Chem.-Biol. Interact..

[81] Filin I.Y., Kitaeva K.V., Rutland C.S., Rizvanov A.A., Solovyeva V.V. (2021). Recent Advances in Experimental Dendritic Cell Vaccines for Cancer. Front. Oncol..

[82] Zimara N., Chanyalew M., Aseffa A., van Zandbergen G., Lepenies B., Schmid M., Weiss R., Rascle A., Wege A.K., Jantsch J. (2018). Dectin-1 Positive Dendritic Cells Expand after Infection with Leishmania major Parasites and Represent Promising Targets for Vaccine Development. Front. Immunol..

[83] Bao M., Ehexige E., Xu J., Ganbold T., Han S., Baigude H. (2021). Oxidized curdlan activates dendritic cells and enhances antitumor immunity. Carbohydr. Polym..

[84] Chen J., Zhao Y., Jiang Y., Gao S., Wang Y., Wang D., Wang A., Yi H., Gu R., Yi Q. (2018). Interleukin-33 Contributes to the Induction of Th9 Cells and Antitumor Efficacy by Dectin-1-Activated Dendritic Cells. Front. Immunol..

[85] Wang F.-S., Agrawal S., Gupta S., Agrawal A. (2010). Human Dendritic Cells Activated via Dectin-1 Are Efficient at Priming Th17, Cytotoxic CD8 T and B Cell Responses. PLoS ONE.

[86] Kim H.S., Park K.H., Lee H.K., Kim J.S., Kim Y.G., Lee J.H., Kim K.H., Yun J., Hwang B.Y., Hong J.T. (2016). Curdlan activates dendritic cells through dectin-1 and toll-like receptor 4 signaling. Int. Immunopharmacol..

[87] Kim S.J., Kim H.S., Seo Y.R. (2019). Understanding of ROS-Inducing Strategy in Anticancer Therapy. Oxidative Med. Cell. Longev..

[88] Yu Y., Shen M., Song Q., Xie J. (2018). Biological activities and pharmaceutical applications of polysaccharide from natural resources: A review. Carbohydr. Polym..

[89] Fatima N., Upadhyay T., Sharma D., Sharma R. (2017). Particulate beta-glucan induces early and late phagosomal maturation in murine macrophages. Front. Biosci..

[90] Jia X., Liu Q., Zou S., Xu X., Zhang L. (2015). Construction of selenium nanoparticles/β-glucan composites for enhancement of the antitumor activity. Carbohydr. Polym..

[91] Fernando W., Rupasinghe H.P.V., Hoskin D.W. (2019). Dietary phytochemicals with anti-oxidant and pro-oxidant activities: A double-edged sword in relation to adjuvant chemotherapy and radiotherapy?. Cancer Lett..

[92] Guo M., Li Y., Lin Z., Zhao M., Xiao M., Wang C., Xu T., Xia Y., Zhu B. (2017). Surface decoration of selenium nanoparticles with curcumin induced HepG2 cell apoptosis through ROS mediated p53 and AKT signaling pathways. RSC Adv..

